# Optogenetic Regulation of Localization and Function of Serotonin Transporter by Modulating Its Interaction with Soluble Guanylate Cyclase

**DOI:** 10.3390/ijms27104587

**Published:** 2026-05-20

**Authors:** Duanbin Tan, Huangjie Ye, Yuting Chen, Xingyu Huang, Xiaoqi Cai, Yuehui Tian, Yuan-Wei Zhang

**Affiliations:** School of Life Sciences, Guangzhou University, Guangzhou 510006, China; 2112314075@e.gzhu.edu.cn (D.T.); 2112414035@e.gzhu.edu.cn (H.Y.); 32214400025@e.gzhu.edu.cn (Y.C.); 1112214006@e.gzhu.edu.cn (X.H.); caixiaoqi@e.gzhu.edu.cn (X.C.); yuehui.tian@gzhu.edu.cn (Y.T.)

**Keywords:** optogenetics, serotonin transporter, serotonin, light-induced dimerization, soluble guanylate cyclase, transporter regulation

## Abstract

Serotonin (5-HT) signaling is strictly controlled by the serotonin transporter (SERT). The present study aims to establish optogenetic approaches for the control of SERT localization and function by modulating the interaction between SERT and its regulatory protein, soluble guanylate cyclase (sGC). We generated several cell lines that stably express blue light-inducible optogenetic elements fused to sGC or the fourth internal loop (IL4) motif of SERT. Our results indicated that blue light-induced SERT-sGC interaction by heterodimerizing SsrA embedded in the membrane-associated improved light-induced dimer (iLID) and SspB-sGCβ1 decreased SERT localization in the plasma membrane, thus reducing the maximum transport velocity of SERT without affecting its *K_m_* for substrate. The light-induced subcellular redistribution of SERT was shown to be attributable to an interference of the SERT-sGC interaction with SERT trafficking but not PKC-mediated internalization. In addition, the light-induced SERT-sGC interaction was blocked by the IL4 peptide or a mutation in the IL4 motif. Furthermore, light-induced exposure of the IL4 motif in iLID decreased the SERT-sGC interaction by displacing SERT from the SERT-sGC complex, thus increasing SERT localization in the membrane and elevating its ability for substrate uptake. This study achieved light-inducible modulation of the protein–protein interaction that allows for the study of biochemical and cellular processes in live cells.

## 1. Introduction

Serotonin transporter (SERT) is a membrane transporter expressed at both the serotonergic neurons in the dorsal raphe nucleus (DRN) and presynaptic terminals in other brain regions, and catalyzes the reuptake of serotonin (5-hydroxytryptamine, 5-HT) from the synaptic cleft. SERT plays a central role in the modulation of 5-HT signaling in the central nervous system (CNS) [1,2,3]. Dysfunction of SERT has been demonstrated to be implicated in the pathophysiology of several psychiatric disorders, such as major depressive disorder (MDD), anxiety, and insomnia [4,5,6]. Importantly, SERT is the primary target of drugs of abuse, such as cocaine and methamphetamines, and antidepressants broadly used for the treatment of MDD, such as the selective serotonin reuptake inhibitors (SSRIs) [7,8]. However, these conventional SSRIs have many shortcomings, such as slow onset, low efficacy, and serious adverse effects, supporting the development of new therapeutic agents or approaches with a novel mechanism of action [9,10].

SERT belongs to the monoamine transporter family, which also includes transporters for dopamine (DAT) and norepinephrine (NET). These monoamine transporters share a common structural feature and similar transport mechanism by which the central binding site is alternately accessible to the extracellular medium or cytoplasm for substrate binding or release by conformational transitions [11,12]. During the past decade, high-resolution structures of SERT bound with its substrate 5-HT or various SSRIs in several conformational states have been resolved, providing a structural basis to understand its transport or inhibition mechanism [13,14,15,16,17]. In addition to the remarkable progress in characterizing the structure and mechanism of SERT, illustrating the regulation of transport function by various biological events can promote us to understand its implications in the pathophysiology of psychiatric disorders or to develop novel targets for effective treatments of 5-HT-related mental diseases [18,19,20].

Recently, two relevant protein components in the cGMP-dependent protein kinase (PKG) signal transduction pathway, neuronal nitric oxide synthase (nNOS), and soluble guanylate cyclase (sGC), have been paid attention to because they have been demonstrated to physically associate with SERT by interacting with different structural motifs in SERT, respectively [21,22]. These protein–protein associations were shown to modulate SERT subcellular distribution and transport activity [21,22]. Most strikingly, disruption of the SERT-nNOS interaction produced fast-onset antidepressant effects in depressive animal models [23,24,25]. These studies have provided new acting targets toward the interactions between SERT and these regulatory proteins for modulating 5-HT signaling.

sGC is a heterodimeric enzyme consisting most commonly of α1 and β1 subunits and catalyzes the conversion of GTP to cGMP upon NO binding to the β1 subunit [26,27]. Our previous results indicated that either α1 or β1 subunit interacts with the fourth internal loop (IL4) in SERT [22]. However, the cellular mechanism by which the SERT-sGC association regulates SERT function remains to be fully understood. Because of low expression levels of both endogenous SERT and sGC in physiologically relevant systems, it is difficult to examine the molecular or cellular process of the transporter in response to various biochemical interventions. On the other hand, specific interaction between the membrane transporter SERT and cytosolic regulatory protein sGC makes them appealing candidates for control through optogenetic approaches. An improved light-inducible dimer (iLID) includes a photosensitive LOV2 domain fused to the SsrA peptide, which can bind to its partner SspB with blue light stimulation [28,29]. In this study, we designed strategies that use iLID to achieve light-inducible modulation of the SERT-sGC interaction in live cells. We expect to create optogenetic tools for studying the cellular process in the SERT-sGC interaction-mediated regulation of SERT function in living systems.

## 2. Results

### 2.1. An Optogenetic Approach for Light-Inducible Interaction Between SERT and sGC

**Figure 1:**
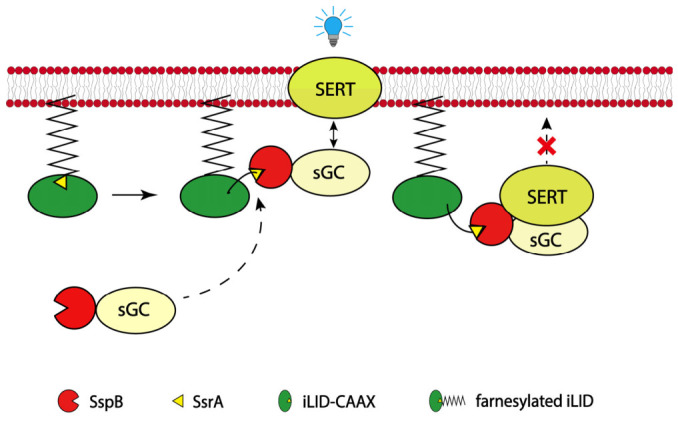
Schematic presentation of light-inducible interaction between SERT and sGC. Under blue light illumination, uncaged SsrA peptide in the membrane-associated iLID binds with SspB fused to the N-terminal tail of sGCβ1. This is proposed to enhance the interaction between sGCβ1 and SERT in the plasma membrane, thus interfering with SERT cellular process.

Transport activity of a solute carrier relies on its expression level in the plasma membrane [30]. To understand the regulation of SERT function by the interaction between SERT and sGC, we employed an optogenetic approach to control the subcellular distribution of sGC (Figure 1). Our previous study indicated that either sGCα1 or β1 subunit interacts with the SERT-IL4 motif and that both sGC subunits share a similar mechanism underlying modulation of the SERT-sGC interaction (22). Theoretically, we could choose either one, α1 or β1, as a representative sGC subunit for the optogenetic manipulation of the SERT-sGC interaction. However, the commercially available antibody for sGCβ1 but not sGCα1 met our experimental requirements; hence, we chose sGCβ1 as a representative subunit for our optogenetic modulation of the SERT-sGC interaction. The N-terminus of sGCβ1 subunit is fused with the SspB adaptor, which is proposed to tightly bind with the SsrA peptide uncaged from the LOV2 domain upon blue light stimulation [28]. The iLID is C-terminal tagged with a peptide sequence (CAAX, A refers to aliphatic amino acids; X represents any amino acid) that anchors the protein to the plasma membrane by farnesylation [31]. The light-inducible binding of the membrane-associated iLID with SspB-sGC is proposed to enhance the interaction between SERT and sGC in the plasma membrane (Figure 1). This light-inducible strategy allows us to examine the cellular process in the regulation of SERT by enhancing the SERT-sGC interaction in live cells.

### 2.2. Light-Inducible Interaction Alters SspB Subcellular Localization

**Figure 2:**
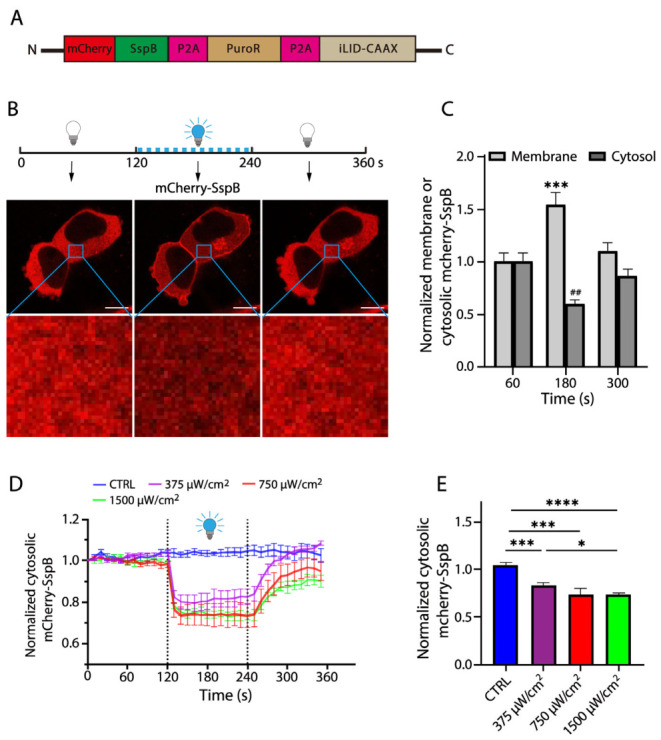
Blue light-inducible interaction between SsrA and SspB. (**A**) A viral construct for generation of HEK 293T stable cells co-expressing mCherry-SspB and iLID-CAAX. PuroR, puromycin resistance; BSDR, blasticidin S resistance; P2A, P2A peptide sequence. (**B**) Schematic diagram of blue light illumination and confocal images. Blue light illumination was performed during a time period from 120 to 240 s. Representative images of mCherry-SspB fluorescence were captured at 60, 180, and 300 s in the light stimulation procedure, respectively. Blue squares represent cytosolic areas selected for measuring fluorescence intensity. Scale bars, 20 μm. (**C**) Subcellular distribution of mCherry-SspB. mCherry fluorescence intensity in the cytosolic or plasma membrane (Figure S2) regions was normalized to that measured in the same regions at the 60 s time point. *** *p* < 0.001, ## *p* < 0.01, compared with fluorescence measured in the cytosolic or plasma membrane areas at the 60 s time point, respectively (*n* = 8). (**D**) Effects of blue light intensity on cytosolic mCherry-SspB fluorescence. mCherry-SspB fluorescence in selected cytosolic areas was monitored over a 360 s time period, in which pulsed blue light was delivered at an intensity of 375, 750, or 1500 μW/cm^2^ in a time period from 120 to 240 s and then normalized to that measured at the 0 s point (*n* = 8). CTRL, control measured without blue light illumination over the entire time period. (**E**) Light intensity-dependent decrease in cytosolic fluorescence of mCherry-SspB. Cytosolic fluorescence of mCherry-SspB under blue light illumination with the indicated blue light intensity at the 180 s point was normalized to that measured in the same areas at the 0 s point. * *p* < 0.05, *** *p* < 0.001, **** *p* < 0.0001, compared with control (*n* = 8). Error bars represent standard error of the mean (SEM). Statistical analyses were performed with a *t*-test between two groups or one-way ANOVA between multiple groups. *n* represents total number of cells used for analyzing fluorescence, with one selected area per cell.

To establish experimental conditions for the optogenetic control of protein–protein interaction, we generated two HEK 293T stable cell lines that express eGFP-iLID-CAAX or mCherry-SspB, respectively (Figure S1A). Confocal imaging analysis indicated that eGFP-iLID was exclusively expressed in the plasma membrane, whereas mCherry-SspB was predominantly localized in the cytosol (Figure S1B). To investigate a blue light-inducible alternation of SspB localization, we generated an additional stable cell line that simultaneously expresses both mCherry-SspB and iLID-CAAX in HEK 293T cells (Figure 2A). To evaluate the light-induced redistribution of mCherry-SspB between the cytosol and plasma membrane, we quantitatively measured a time-dependent alteration of mCherry fluorescence in randomly selected cytosolic areas or plasma membrane regions of the cells with or without blue light stimulation (Figure 2B and Figure S2). As shown in Figure 2C and Figure S2, compared to the control at the 60 s time point, blue light stimulation for 60 s (180 s time point) led to a significant recruitment of the mCherry-SspB fluorescence in the plasma membrane, which could be recovered to the levels comparable to those in the control after blue light was eliminated for 60 s (300 s time point). Because the content of a reduction in the cytosolic fluorescence matched well with that of an increase in the membrane fluorescence in our experimental settings, we used an alteration of the cytosolic fluorescence as a standard to assess the light-inducible redistribution of a cytosolic protein (Figure 2B,C). We then examined the effects of various blue light intensities on the cytosolic fluorescence of mCherry-SspB. Our results showed that the cytosolic fluorescence of mCherry-SspB decreased in a blue light intensity-dependent manner (Figure 2D). Exposure to a pulsed blue light (2.5 s pulse duration every 10 s) at 1500 μW/cm^2^ for a total of 2 min resulted in an approximate 30% reduction in the cytosolic fluorescence of mCherry-SspB (Figure 2E).

### 2.3. Light-Inducible Translocation of sGC to the Plasma Membrane

**Figure 3:**
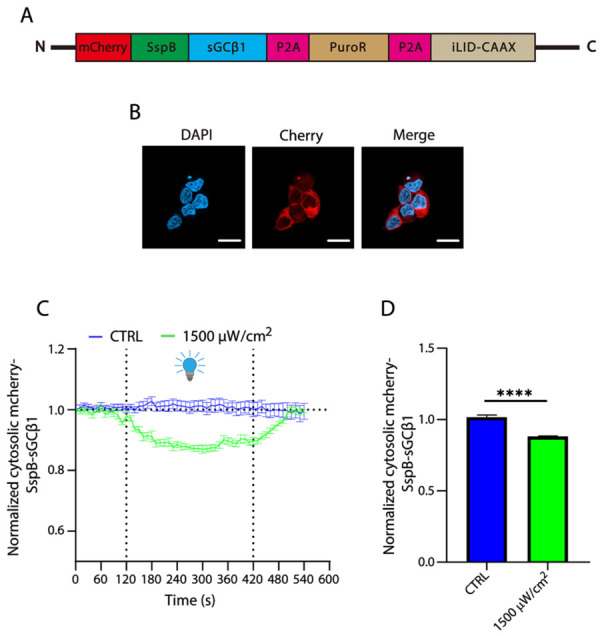
Light-induced decrease in cytosolic SspB-sGCβ1 fluorescence. (**A**) A viral construct for generation of HEK 293T stable cells co-expressing mCherry-SspB-sGCβ1 and iLID-CAAX. (**B**) Representative confocal images of mCherry-SspB-sGCβ1. Scale bars, 20 μm. (**C**) Time course of cytosolic mCherry-SspB-sGCβ1 fluorescence in a 540 s light stimulation procedure. Blue light at 1500 μW/cm^2^ was delivered from 120 to 420 s. CTRL, control measured without light illumination (*n* = 8). (**D**) Light-induced decrease in cytosolic mCherry-SspB-sGCβ1 fluorescence. Cytosolic fluorescence of mCherry-SspB at the 300 s point was normalized to that measured in the same areas at the 0 s point. **** *p* < 0.0001 (*n* = 8). Error bars represent SEM. Statistical analysis was performed with *t*-test between two groups in (**D**). *n* represents total number of cells used for analyzing fluorescence, with one selected area per cell.

To observe light-inducible redistribution of sGC, we generated a stable cell line that simultaneously expresses sGC with mCherry-SspB fused in the N-terminus and iLID-CAAX (Figure 3A). In the absence of blue light illumination, mCherry-SspB-sGC was exclusively expressed in the cytosol (Figure 3B). A blue light stimulation procedure at 1500 μW/cm^2^ for 2 min did not effectively induce sGC translocation to the plasma membrane (Figure S3). Thus, we illuminated the stable cells to initiate mCherry-SspB-sGC translocation for an extended period. As shown in Figure 3C,D and Figure S4 (confocal images), a 5 min light stimulation resulted in a small but significant decrease (approximately 15%) in the cytosolic fluorescence, indicating a translocation of sGC to the plasma membrane. We further increased light stimulation to 10 min, but a significant difference in sGC translocation was not observed, compared to that with a 5 min stimulation. We assume that the light-induced translocation of sGCβ1 to the plasma membrane may reach saturation after 5 min of stimulation, thus leading to stable fluorescence.

### 2.4. Optogenetic Regulation of SERT Function by Enhancing the SERT-sGC Interaction

To learn if blue light illumination produces direct effects on the cells or SERT function, we performed cell counting kit-8 (CCK-8, New Cell & Molecular Biotech, Suzhou, China) and transport assays with cells stably expressing SERT without the optogenetic elements in the absence or presence of a blue light illumination procedure. Our results indicated that a 5 min illumination at 1500 μW/cm^2^ had little effect on cell viability and 4-(4-(dimethylamino)phenyl)-1-methylpyridinium (APP^+^) uptake (Figure S5A,B), suggesting that the illumination procedure did not damage the cells or SERT.

**Figure 4:**
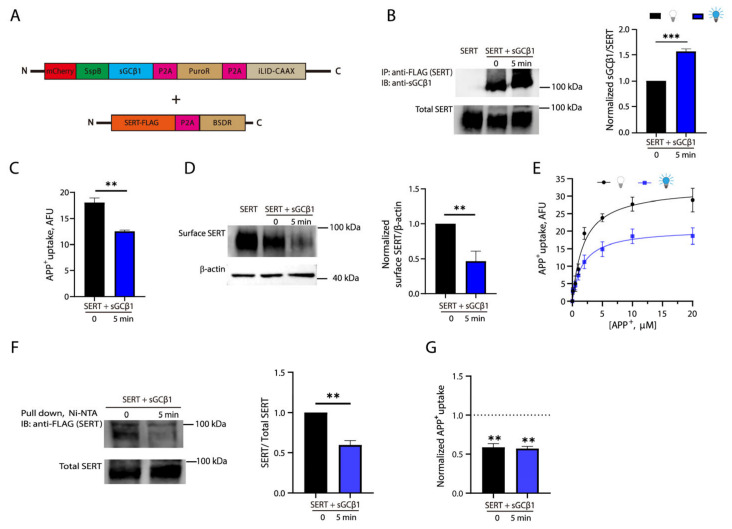
Light-induced SERT-sGC interaction decreased transport activity of SERT by reducing its cell surface localization. (**A**) Viral constructs for generation of HEK 293T stable cells co-expressing SERT-FLAG, mCherry-SspB-sGCβ1, and iLID-CAAX with a double-resistance to puromycin and blasticidin S. (**B**–**E**) Effects of blue light illumination on the SERT-sGC interaction, transport activity, cell surface localization, and transport kinetics. HEK 293T stable cells expressing SERT-FLAG or co-expressing SERT-FLAG/mCherry-SspB-sGCβ1/iLID-CAAX were illuminated by blue light at 1500 μW/cm^2^ for 5 min, and immediately subjected to immunoprecipitation (**B**), transport assay (**C**), biotinylation (**D**), or kinetic analysis (**E**) as described under Section 4. These biochemical analyses were also performed in parallel with the cells without blue light illumination. For the transport assay, APP^+^ uptake was determined with triplicate measurements in each experiment. AFU, arbitrary fluorescence units. In co-immunoprecipitation analysis, sGCβ1 immunoreactivity in anti-FLAG immunoprecipitates was normalized to the expression level of total SERT. In biotinylation analysis, SERT expression on the cell surface was expressed as an immunoreactivity of biotinylated SERT normalized to an immunoreactivity of a housekeeping protein, β-actin. In kinetic analysis for APP^+^ uptake, K_m_ and V_max_ values represent mean ± SEM from three experiments. (**F**) Effect of the SERT-sGC association on the interaction between SERT and SEC24. Pull-down assay was performed by incubating Ni-NTA beads with cell lysates from the cells co-expressing SERT-FLAG, opto-sGC, and SEC24-His_6_ after being treated with or without blue light illumination for 5 min. SERT-FLAG in imidazole eluates was detected by immunoblot with anti-FLAG antibody. SERT-FLAG immunoreactivity in imidazole eluates was normalized to the expression level of total SERT. (**G**) Effect of blue light illumination on the PKC-mediated reduction in transport activity. HEK 293T cells stably co-expressing SERT-FLAG and opto-sGC were pre-incubated with vehicle or 1 μM β-PMA for 1 h and then treated with or without blue light illumination for an additional 5 min. The cells were then used for a transport assay as described under Section 4. APP^+^ uptake was normalized to that measured with the control cells without β-PMA treatment, shown as a dotted line., ** *p* < 0.01, *** *p* < 0.001. Error bars represent SEM from three independent experiments (*n* = 3). Statistical analysis was performed with a *t*-test between two groups.

We then performed co-transfection with an additional viral plasmid encoding C-terminal FLAG-tagged SERT in the HEK 293T cell line shown in Figure 3A and selected the colonies with a double-antibiotic (puromycin and blasticidin S) resistance (Figure 4A). The cells were used to examine the effects of blue light illumination on the SERT-sGC interaction by co-immunoprecipitation (Figure 4B). In the absence of blue light illumination, sGCβ1 immunoreactivity was detected in anti-FLAG immunoprecipitates, confirming a naturally occurring interaction between SERT and sGC in the cells co-expressing SERT and sGC. By comparison, exposure to blue light for 5 min resulted in an increase of approximately 50% in sGCβ1 immunoreactivity in anti-FLAG immunoprecipitates, indicating that blue light stimulation remarkably increased the SERT-sGC association. Notably, this blue light illumination procedure did not alter total SERT expression. The SERT-sGC complex at the plasma membrane can be dynamically internalized by endocytosis, a basic cellular process. Our co-immunoprecipitation experiments were performed with total cell lysates, which included both the SERT-sGC complex at the plasma membrane and the internalized complex in the cytosolic compartment. On the other hand, the decrease in the cytosolic SspB-sGCβ1 fluorescence reflects a net change in the cytosolic SspB fluorescence, including a decrease caused by the light-induced cytosol-to-membrane translocation and an increase due to the naturally occurring internalization. These measurements led to a small decrease in the cytosolic SspB-sGC fluorescence measured with the live cells (Figure 3D), compared to a large increase in the SERT-sGC association estimated with the cell lysates (Figure 4B). Nevertheless, our experiments demonstrated that these optogenetic tools can be used for investigating the cellular process of the transporter protein in response to an enhancement of the SERT-sGC association.

The cells were then used to examine the effects of blue light illumination on their capability for the uptake of a fluorescent substrate, APP^+^. As shown in Figure 4C, compared to the control cells without light stimulation, a 5 min blue light illumination procedure led to a significant decrease (~ 33%) in the capability of cells for the uptake of APP^+^, suggesting that a blue light-induced enhancement of the SERT-sGC interaction reduced SERT activity. Next, we performed biotinylation of the membrane proteins by using a membrane-impermeant biotinylation reagent sulfo-NHS-SS-biotin to investigate the effects of blue light illumination on SERT expression on the cell surface (Figure 4D). Compared to the control, blue light illumination for 5 min led to a profound decrease in SERT expression on the cell surface. Moreover, we performed kinetic analysis for APP^+^ uptake to further evaluate the effects of the SERT-sGC interaction on SERT function. *K_m_* and *V_max_* values were 1.92 ± 0.32 μM and 33.50 ± 3.68 AFU/mg/min without light illumination, respectively, while 1.70 ± 0.43 μM and 20.99 ± 1.99 AFU/mg/min with light illumination (Figure 4E). Statistical analysis indicated that blue light stimulation remarkably decreased *V_max_* value for APP^+^ uptake (*p* < 0.05) but with little effect on *K_m_* for the substrate, suggesting that an impaired transport activity is attributable to a decreased cell surface expression of SERT rather than an alteration of its catalytic function.

Next, we asked what cellular process responsible for subcellular redistribution of the transporter protein mediated by the SERT-sGC interaction. To answer this question, we examined the effects of blue light-induced SERT-sGC interaction on the association of SERT with SEC24C, which is an essential component of the coat protein complex II that plays a critical role in SERT membrane trafficking by binding with SERT [32,33]. The double-resistant stable cells co-expressing SERT-FLAG and opto-sGC were transiently transfected with a plasmid encoding C-terminal 6 x His-tagged SEC24C (SEC24C-His_6_) and then treated with or without blue light illumination for 5 min. The cells were lysed, and the resulting lysates were immediately incubated with Ni-NTA beads. The imidazole eluates were then analyzed by immunoblot with anti-FLAG antibody. As shown in Figure 4F, under the control conditions, SERT-FLAG immunoreactivity was detected in the eluates from Ni-NTA beads, confirming a basal SERT-SEC24C association. By comparison, blue light stimulation significantly reduced immunoreactivity of SERT-FLAG, suggesting that blue light-induced enhancement of the SERT-sGC interaction dissociates SEC24C from SERT. To see if the SERT-sGC interaction affects PKC-mediated reduction in transport activity due to SERT internalization, we examined the effects of blue light illumination on APP^+^ uptake in the absence or presence of a potent PKC activator, β-phorbol 12-myristate 13-acetate (β-PMA). The cells stably co-expressing SERT-FLAG and opto-sGC were pre-incubated with vehicle or β-PMA and then treated with or without blue light illumination. Our transport assay results indicated that activation of PKC with β-PMA remarkably reduced SERT capability for the uptake of substrate (~ 45%) in the absence of blue light illumination (Figure 4G), consistent with previous works [34,35]. However, a 5 min blue light illumination procedure did not lead to a statistically significant alteration of β-PMA-induced decrease in APP^+^ uptake, suggesting that the SERT-sGC interaction has little effect on PKC-mediated internalization of SERT.

As control experiments, we examined total expression of SERT or sGCβ1 under our experimental conditions, although the stable cell lines were used for all experiments in our study. As shown in Figure S6, the total expression level of neither SERT nor sGCβ1 was altered by light illumination. In addition, we also examined the effects of the light illumination-induced SERT-sGC association on sGC activity. sGC catalyzes the production of cGMP upon NO binding to the sGCβ1 subunit; however, its activity requires the coexistence of two subunits and the formation of a heterodimer sGCα1/β1. It has been demonstrated that expression of a single subunit alone, sGCα1 or sGCβ1, did not produce an active sGC [36,37]. To confirm it in our optogenetic expression systems, we assessed sGC activity by measuring cGMP production in HEK 293T cells under the experimental conditions in the absence or presence of a sGC stimulator, *S*-nitroso-*N*-acetyl penicillamine (SNAP), which breaks down spontaneously to produce NO in an aqueous medium. Unfortunately, cGMP levels in the absence of SNAP were below the detection limit. By contrast, the addition of SNAP significantly stimulated sGC activity for cGMP production. However, exposure to light illumination had little effect on cGMP production in our expression system, either expressing SERT alone or co-expressing SERT with opto-sGCβ1 (Figure S5C), although the light illumination procedure has been indicated to enhance the SERT-sGCβ1 association.

### 2.5. A Mutation in SERT-IL4 Disrupts Blue Light-Induced SERT-sGC Interaction

**Figure 5:**
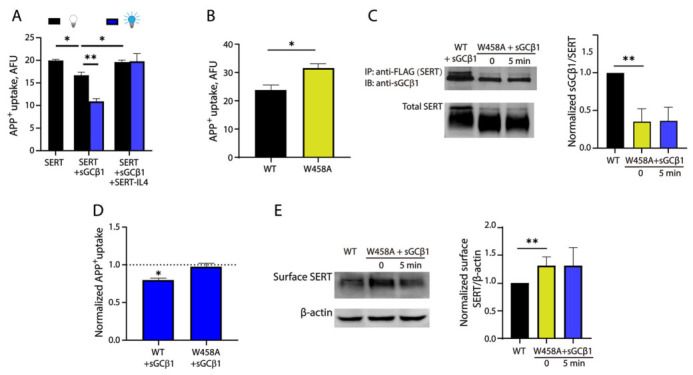
The SERT-sGC interaction was disrupted by a mutation in the SERT-IL4 motif. (**A**) Effect of the SERT-IL4 peptide on transport activity. HEK 293T stable cells expressing SERT-FLAG or co-expressing SERT-FLAG/mCherry-SspB-sGCβ1/iLID-CAAX were pretreated with vehicle or a synthesized SERT-IL4 peptide fused to the C-terminal tail of TAT (1 μM) in culture medium at 37 °C for 3 h and then treated with or without blue light illumination at 1500 μW/cm^2^ for an additional 5 min. The cells were immediately subjected to transport assay. (**B**) Effect of W458A mutation in the IL4 motif on transport activity. HEK 293T cells stably expressing SERT WT or W458A mutants were used for analyzing its transport activity. (**C**–**E**) W458A mutation attenuated blue light-induced alterations of the SERT-sGC interaction, transport activity, and cell surface expression. HEK 293T cells stably expressing SERT-FLAG or co-expressing SERT-FLAG/mCherry-SspB-sGCβ1/iLID-CAAX or W458A-FLAG/mCherry-SspB-sGCβ1/iLID-CAAX were treated with or without blue light illumination at 1500 μW/cm^2^ for 5 min and then subjected to immunoprecipitation (**C**), transport assay (**D**), or biotinylation (**E**), respectively, as described under Section 4. In the transport assay, a dotted line represents transport activity of WT or W458A mutant without blue light illumination. In co-immunoprecipitation analysis, sGCβ1 immunoreactivity in anti-FLAG immunoprecipitates was normalized to the expression level of total SERT. In biotinylation analysis, SERT expression on the cell surface was expressed as an immunoreactivity of biotinylated SERT normalized to an immunoreactivity of β-actin. * *p* < 0.05, ** *p* < 0.01,). Error bars represent SEM from three independent experiments (*n* = 3). Statistical analysis was performed with a *t*-test between two groups.

Our previous study identified the IL4 motif connecting transmembrane domains 8 and 9 in SERT that plays a key role in the interaction with sGC [22]. To examine the effects of the IL4 motif on the transport activity of SERT, we treated the cells with a synthesized IL4 peptide fused with a cell-penetrating peptide TAT (TAT-IL4) for 3 h in the culture medium and then performed a transport assay for APP^+^ uptake. As shown in Figure 5A, IL4 treatment not only effectively attenuated the basal SERT-sGC interaction-induced reduction in APP^+^ uptake (~17%) but also reversed the blue light-induced decrease in transport activity (~30%). The Trp458 residue in SERT-IL4 is unique based on a sequence alignment of the IL4 motifs in all three monoamine transporters [38]. To explore the role of Trp458 in the SERT-sGC interaction, we substituted Trp458 with alanine and examined the effects of W458A mutation on SERT expression and function. Compared to WT, the transport activity of the W458A mutant was increased by approximately 22% (Figure 5B), indicating the mutation led to an elevation of its basal transport function. When co-expressed with opto-sGCβ1 components, our co-immunoprecipitation experiments showed that sGCβ1 immunoreactivity was remarkably lower with W458A mutant in anti-FLAG immunoprecipitates than that with WT SERT (Figure 5C). Importantly, immunoreactivity of W458A was not responsive to blue light stimulation, indicating that Trp458 is critical for the IL4-mediated SERT-sGC interaction. In addition, our transport assay results showed that a 5 min blue light stimulation significantly decreased transport activity of WT by approximately 20% but had little effect on the capability of W458A for APP^+^ uptake (Figure 5D). Furthermore, our biotinylation experiments indicated that substitution of Trp458 with Ala enhanced SERT cell surface expression level by approximately 1.25-fold, consistent with an increase in transport activity with the W458A mutant (Figure 5E). Notably, the cell surface expression of W458A was no longer sensitive to blue light illumination, compared to WT. These results suggest that an impaired SERT-sGC interaction in the W458A mutant results in an increase in its cell surface expression, thereby enhancing its transport activity.

### 2.6. Optogenetic Regulation of SERT Function by Interfering with the SERT-sGC Interaction

**Figure 6:**
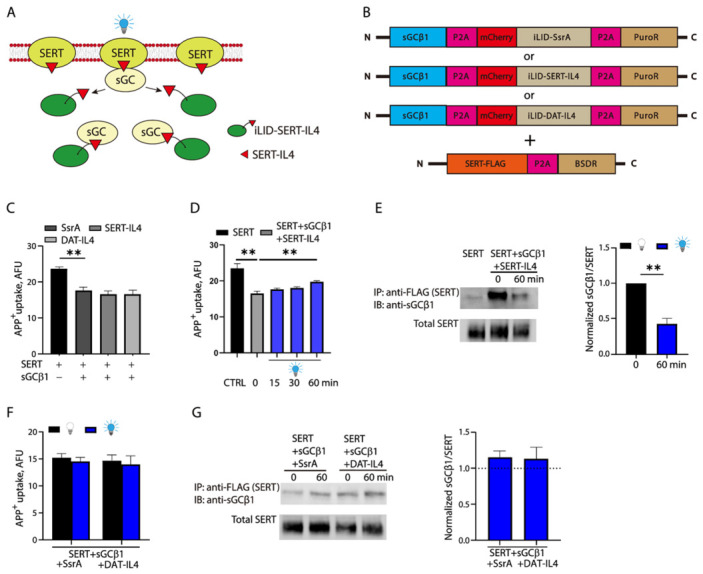
Light-inducible disruption of the SERT-sGC interaction by SERT-IL4 increases transport activity of SERT. (**A**) A strategy for light-inducible disruption of the SERT-sGC interaction. SERT-IL4 peptide is proposed to be buried within iLID in the absence of blue light illumination. When applied for blue light stimulation, iLID uncages SERT-IL4, which prevents cytosolic sGC from interacting with SERT or displaces SERT from the SERT-sGC complex. (**B**) Constructs for generation of HEK 293T stable cell lines co-expressing SERT-FLAG, sGCβ1, and iLID-SsrA, SERT-IL4, or DAT-IL4 with a double-resistance to puromycin and blasticidin S. (**C**) Transport assay without blue light illumination. HEK 293T cells stably expressing SERT-FLAG or co-expressing SERT-FLAG/sGCβ1/iLID-SsrA, SERT-IL4, or DAT-IL4 were used for measuring their capabilities for APP^+^ uptake in the absence of blue light illumination as prescribed under Section 4. (**D**) Transport assay with blue light illumination. HEK 293T cells stably co-expressing SERT-FLAG/sGCβ1/iLID-SERT-IL4 were treated with or without blue light illumination at 1500 μW/cm^2^ for the indicated time periods and then subjected to transport assay. CTRL, control cells stably expressing SERT-FLAG without blue light illumination. (**E**) Co-immunoprecipitation. HEK 293T cells stably co-expressing SERT-FLAG/sGCβ1/iLID-SERT-IL4 were treated with or without blue light illumination for 60 min and then subjected to immunoprecipitation with anti-FLAG antibody. sGCβ1 immunoreactivity in anti-FLAG immunoprecipitates was normalized to the expression level of total SERT. (**F**,**G**) Effect of iLID-SsrA or DAT-IL4 on transport activity and the SERT-sGC interaction. HEK 293T cells stably co-expressing SERT-FLAG/sGCβ1/iLID-SsrA or DAT-IL4 were treated with or without blue light illumination for 60 min and then subjected to transport assay (**F**) and co-immunoprecipitation (**G**). In co-immunoprecipitation, sGCβ1 immunoreactivity in anti-FLAG immunoprecipitates was normalized to the expression level of total SERT. A dotted line represents normalized sGCβ1/SERT without blue light illumination. ** *p* < 0.01. Error bars represent SEM from three independent experiments (*n* = 3). Statistical analysis was performed with a *t*-test between two groups.

Our data indicated that the SERT-IL4 motif modulates SERT localization and function by mediating the SERT-sGC interaction. We consider whether an optogenetic approach can be used to manipulate SERT-IL4 so as to interfere with the SERT-sGC interaction. To this end, we designed a strategy for the optogenetic control of SERT-IL4 exposure by inserting it into iLID, in which the C-terminal prenylation motif is removed (Figure 6A). Availability of the SERT-IL4 motif to interact with sGC depends on blue light illumination. We generated several double-antibiotic-resistant cell lines co-expressing C-terminal FLAG-tagged SERT, sGCβ1, and iLID with an inserted SsrA, SERT-IL4, or DAT-IL4, respectively (Figure 6B). Our transport assay showed that co-expression of iLID-SsrA, SERT-IL4, or DAT-IL4 did not alter sGCβ1-induced decrease (~32%) in transport activity of SERT, suggesting that the SERT-IL4 motif was caged within iLID in the absence of blue light illumination (Figure 6C). By contrast, under blue light stimulation, SERT-IL4 significantly attenuated sGCβ1-induced decrease in SERT activity by restoring ~80% capability for APP^+^ uptake in a time-dependent manner, although it did not completely reverse the effects of sGCβ1 in the time periods tested (Figure 6D). After exposure to blue light illumination for 60 min, cells were lysed, and the resulting lysates were then subjected to co-immunoprecipitation using an anti-FLAG antibody. Our data indicated that blue light stimulation remarkably reduced sGCβ1 immunoreactivity in FLAG immunoprecipitates by more than 50%, suggesting that blue light activated iLID to uncage the SERT-IL4 motif for competitive displacement of SERT in the SERT-sGCβ1 complex, thereby impairing the SERT-sGC interaction (Figure 6E). On the other hand, the same blue light illumination procedure had little effect on both SERT activity and the SERT-sGC interaction when co-expressed with iLID-SsrA or DAT-IL4 (Figure 6F,G), indicating a specificity for the IL4-mediated SERT-sGC interaction.

## 3. Discussion

The present study established optogenetic approaches for the regulation of SERT function by modulating the interaction between SERT and its regulatory protein sGC. Our results indicated that the light-induced SERT-sGC interaction by heterodimerizing SsrA and SspB decreased SERT localization in the plasma membrane, thereby reducing its transport activity. By contrast, light-induced exposure of the IL4 motif in iLID decreased the SERT-sGC interaction by competitive displacement, thus increasing SERT localization in the plasma membrane and elevating its capability for substrate uptake. By modulating the SERT-sGC interaction with these optogenetic tools, we achieved either up- or down regulation of SERT function.

SERT on the cell surface is proposed to be present in a dynamic equilibrium, which is regulated by the exocytosis and endocytosis of the transporter protein [39,40]. Alteration of these essential cellular processes has been believed to affect the equilibrium, which, in turn, alters transporter distribution on the cell surface [40,41]. The C-terminal region of SERT contains the critical residues that form a docking site for the SEC23/SEC24C complex, which is the core component of the coat protein complex II that forms vesicles for membrane trafficking of the transporter protein [32,33]. Our results demonstrated that blue light-induced enhancement of the SERT-sGC association decreased the interaction between SERT and SEC24C. Thus, we propose that light-induced SERT-sGC interaction produces a spatial barrier that prevents the C-terminal residues from binding with SEC23/SEC24C, thereby leading to impaired membrane trafficking and decreased SERT localization on the cell surface. When the SERT-sGC interaction is reduced by light-induced displacement with the IL4 motif, we propose that SERT trafficking from the ER to the plasma membrane is promoted, resulting in an increased SERT distribution on the cell surface.

In the present study, two optogenetic strategies have been used to increase or decrease the SERT-sGC interaction. For increasing the SERT-sGC interaction, our strategy was to recruit sGC to the plasma membrane by the binding of SsrA embedded in the membrane-associated iLID with SspB fused to sGCβ1 upon blue light stimulation, thereby increasing its opportunity to interact with the membrane transporter. This strategy can avoid N- or C-terminal modification of SERT with bulky optogenetic elements. Either N- or C-terminus of SERT contains essential sequence elements for post-translational modifications, such as phosphorylation [42,43,44,45,46] and ubiquitination [47,48], and for docking of components of the endoplasmic reticulum (ER) export machinery [49,50]. Therefore, it is reasonable to speculate that N- or C-terminal modification by either iLID or SspB might produce lethal effects on the cellular process of SERT. For decreasing the SERT-sGC interaction, we replaced the SsrA (6 amino acids, NDENYF) with the SERT-IL4 (8 amino acids, FPHVWAKR) in iLID without the C-terminal tetrapeptide motif (CAAX), yielding a fully cytosolic protein. Our results indicated that the IL4 motif was well buried within iLID but uncaged upon blue light illumination. This light-inducible property renders an ability for iLID-SERT-IL4 to reduce the SERT-sGC interaction by competitive displacement under blue light stimulation.

SERT, through its reuptake activity, plays a key role in 5-HT homeostasis in the CNS [51,52]. Dysregulation of 5-HT level has been demonstrated to be a critical pathological factor in several psychiatric disorders [53,54,55]. Previous studies have revealed several naturally occurring SERT coding variants that alter the transporter regulation by PKG and p38 MAPK-linked pathways and influence risk for these disorders attributed to compromised 5-HT signaling [55,56,57,58]. In addition to the effects of the assumed phosphorylation, it is worth investigating if these variants alter SERT subcellular localization by interfering with its interaction with the protein components in these signaling cascades. Application of light-inducible protein–protein interaction allows for the spatial and temporal control of a variety of biochemical and cellular processes in living systems [59,60,61,62,63]. Here, we were able to control SERT activity by modulating its localization through an optogenetic manipulation of the SERT-sGC interaction. We expect that an optogenetic strategy would provide a powerful tool to illustrate the molecular mechanism by which the regulation of SERT function is altered by the regulatory proteins in the physiopathology of 5-HT-related mental disorders.

Most importantly, rebalancing of 5-HT levels has become a prominent target in the treatment of 5-HT-related mental disorders [64,65,66]. Administration of the SSRI antidepressants elevates 5-HT level in the DRN by inhibiting somatodendritic SERT, thus resulting in a decrease in 5-HT release in the cortex and the hippocampus due to an activation of somatodendritic 5-HT_1A_ autoreceptors [67]. The SSRIs exert antidepressant effects only after 5-HT_1A_ autoreceptors are desensitized for several weeks, thereby resulting in a delayed onset of the conventional SSRI antidepressants [68,69]. A recent study has demonstrated that disrupting the interaction between SERT and its regulatory protein, such as nNOS, produced rapid-onset antidepressant effects by decreasing 5-HT levels in the DRN and consequently inactivating 5-HT_1A_ autoreceptors and increasing 5-HT release in the cortex and the hippocampus [23]. This study achieved a rapid, regional regulation of 5-HT signaling by modulating the interaction between SERT and nNOS and consequently opened up a novel target for developing fast-acting antidepressants targeting the protein–protein interaction rather than the transporter itself. The present study explored the optogenetic modulation of SERT localization and activity at the molecular and cellular levels. Further study to investigate the effects of the optogenetic modulation on the interaction between SERT and sGC in animal models is required for establishing 5-HT-related depressive animal models by optogenetic enhancement of the SERT-sGC interaction or developing optogenetic therapeutics by optogenetic dissociation of sGC from SERT in the treatment of 5-HT-related psychiatric disorders.

## 4. Materials and Methods

### 4.1. Materials

HEK 293T cells were from the American Type Culture Collection (Manassas, VA, USA). APP^+^ and protease inhibitor mixture cocktail were purchased from Sigma-Aldrich (Burlington, MA, USA). Anti-FLAG agarose gel, streptavidin agarose, BCA protein assay kit, lipofectamine 2000, and super signal West pico were from ThermoFisher Scientific (Waltham, MA, USA). Anti-sGCβ1 was purchased from Santa Cruz Biotechnology (Dallas, TX, USA). All other reagents were of analytical grade.

### 4.2. Lentiviral Plasmids

All plasmids were constructed by PCR-based amplification of the gene sequences and subsequently by inserting them into a lentiviral plasmid using Ezmax Universal CloneMix (Tolobio, Shanghai, China). For generating a plasmid encoding mCherry-SspB-sGCβ1, a flexible GSG linker sequence was introduced between mCherry-SspB and sGCβ1. For generating a plasmid co-expressing multiple genes, a P2A self-cleaving sequence was placed between two gene sequences. As negative controls for photobleaching correction, constructs lacking the iLID component, such as mCherry-SspB or mCherry-SspB-sGC, were also generated, respectively. The lentiviral plasmid, Lenti-EF-1α-SERT-BSD encoding SERT-FLAG, was used as described previously [70]. In addition, a lentiviral plasmid for mCherry-iLID-SERT-IL4 and its control constructs for mCherry-iLID-DAT-IL4 or mCherry-iLID-SsrA were generated, respectively, to examine the specificity for the SERT-sGC interaction.

### 4.3. Mutagenesis and Stable Cell Line Generation

The lentiviral plasmid encoding C-terminal FLAG-tagged SERT was used as a template for mutagenesis. SERT W458A mutant was generated using the Mut Express II Fast Mutagenesis Kit (Vazyme, Nanjing, China) and confirmed by full-length DNA sequencing.

Lentivirus was prepared by transfecting a mixture of a lentiviral plasmid and two other packaging vectors, psPAX2 and pMD2G, into HEK 293T cells, as described previously [71]. HEK 293T cells were then infected by the lentivirus with polybrene in Dulbecco’s Modified Eagle’s Medium (DMEM) with 12 µg/mL blasticidin S, 2 µg/mL puromycin, or both. The culture medium was changed every three days until colonies of resistant cells were formed. Cell lysates were prepared from confluent cultures and subjected to immunoblot analysis to confirm expression of the proteins of interest. The cells were then maintained in DMEM supplemented with 10% fetal bovine serum, 100 units/mL penicillin, 100 µg/mL streptomycin, and 12 µg/mL blasticidin S, 2 µg/mL puromycin, or both at 37 °C in a humidified 5% CO_2_ incubator.

### 4.4. Live Cell Imaging and Membrane Translocation Assay

Time-lapse images were acquired using a laser scanning confocal microscope system configured for upright imaging. The system, composed of a Zeiss LSM 900 scanning unit (Zeiss, Oberkochen, Germany) with Airy scan 2 super-resolution detection, was mounted on a Zeiss Axio Imager Z2 microscope (Zeiss, Oberkochen, Germany). Two solid-state lasers (488 nm and 561 nm) equipped in the microscope were used for photoactivation and excitation of the mCherry fluorophore, respectively, during optogenetic stimulation and imaging. A high-numerical-aperture Plan-Apochromat 40×/1.3 oil immersion objective lens (Zeiss, Oberkochen, Germany) was used for all experiments.

Membrane translocation assay was performed with HEK 293T cells stably expressing optogenetic elements. The assay began under dark conditions with a baseline image acquired using only the 561 nm laser to capture the pre-stimulation localization of mCherry-SspB or mCherry-SspB-sGC. Then, the entire cell area was stimulated by scanning with the 488 nm laser at varying intensities, with each stimulation scan delivering a total illumination period of 2.5 s. Immediately following each stimulation scan, the same region was scanned with the 561 nm laser to acquire an image for monitoring the resultant translocation of mCherry-SspB or mCherry-SspB-sGC. This paired order—a 2.5 s 488 nm stimulation scan followed by a 7.5 s 561 nm imaging—constituted a single measurement cycle. Cycles were repeated every 10 s throughout the experiment to generate time-lapse images.

### 4.5. Image Analysis

All images were analyzed by ZEN 3.10 (Zeiss, Oberkochen, Germany) and ImageJ 1.54k (NIH, Bethesda, MD, USA). Graphical presentations were generated with GraphPad Prism 8 (GraphPad, Boston, MA, USA).

Membrane translocation of mCherry-SspB or opto-sGCβ1 was evaluated by quantifying changes in cytosolic fluorescence [72,73,74]. The fluorescence intensity was measured within a defined cytosolic region (4.00 μm × 4.00 μm) throughout the entire imaging procedure. After background subtraction, the relative fluorescence level was calculated by normalizing to the intensity at time 0 s. To correct non-specific photobleaching, a parallel analysis was performed with the control cells lacking the iLID component. The fluorescence decay profile obtained from the control cells was then subtracted from the experimental data to yield the final corrected translocation signals.

To analyze the temporal changes in fluorescence intensity at the plasma membrane, a line-scan analysis was performed [74]. A straight line was drawn traversing the cell body in each image. The fluorescence intensity profile along this line was measured for every time point throughout the imaging procedure. The position of the plasma membrane on this line was identified by comparing the intensity profiles before and after blue light stimulation, specifically by detecting the characteristic peaks corresponding to the plasma membrane. The fluorescence intensity values at these membrane-associated pixel positions were then extracted and plotted over time to quantify the dynamics of membrane-localized fluorescence.

### 4.6. Light Stimulation

Optogenetic stimulation to examine the SERT-sGC interaction was performed using a self-built programmable blue light device with a peak emission wavelength at 450 nm. The light intensity could be precisely adjusted by an Arduino UNO R4 microcontroller (Arduino S.r.l., Monza, Italy). The output intensity was measured and calibrated using an LH-137 blue light photometer (Lianhc, Shenzhen, China).

Cells were dark-adapted for 24 h and then transferred to a light-tight chamber, followed by light illumination with programmed cycles. Each cycle lasted 10 s and consisted of a 2.5 s pulse of blue light at 1500 μW/cm^2^ with a 7.5 s dark interval.

### 4.7. APP^+^ Uptake Assay

Cells stably expressing SERT or co-expressing SERT and opto-sGCβ1 were illuminated by blue light in the programmable LED light box or kept in the dark for the indicated periods. After light stimulation, cells were immediately used for measuring SERT activity by incubating with 2 μM APP^+^ in KRH buffer containing 20 mM HEPES, pH 7.4, 120 mM NaCl, 1.3 mM KCl, 2.2 mM CaCl_2_, 1.2 mM MgSO_4_, and 0.1% (*w*/*v*) glucose at 22 °C for 5 min. The reaction was stopped by three rapid washes with ice-cold phosphate-buffered saline (PBS, 137 mM NaCl, 2.7 mM K_2_SO_4_, 4.3 mM Na_2_HPO_4_, and 1.4 mM KH_2_PO_4_, pH 7.4), and APP^+^ accumulation in the cells was measured with an Infinite 200 Pro microplate reader (Tecan, Männedorf, Switzerland) as described previously [75]. The excitation wavelength for APP^+^ was 488 nm, while the emission filter was set at 525 nm. Nonspecific APP^+^ uptake was determined in the presence of 1 mM fluoxetine. For kinetic analysis for APP^+^ uptake, cells stably co-expressing SERT and opto-sGCβ1 grown in 96-well plates to full confluence were treated with or without blue light illumination for total 5 min including a 2.5 s pulse of blue light at 1500 μW/cm^2^ with a 7.5 s dark interval in every 10 s, and then incubated with a range of APP^+^ concentrations (0–20 μM) in KRH buffer at 22 °C for an additional 5 min. After washing with ice-cold PBS three times, APP^+^ accumulated in the cells was measured with an Infinite 200 Pro microplate reader (Tecan, Männedorf, Switzerland).

### 4.8. Co-Immunoprecipitation

Co-immunoprecipitation was performed as described previously [76]. Briefly, cells were lysed in RIPA buffer (50 mM Tris-HCl, pH 8.0, 150 mM NaCl, 1 mM EDTA, and 1% Triton X-100) supplemented with protease inhibitor cocktail, and the cell lysates were separated by centrifugation at 15,000× *g* for 20 min at 4 °C. The resulting supernatants were then incubated with anti-FLAG agarose beads at 4 °C overnight with gentle rotation. The next day, the beads were collected and washed three times with ice-cold RIPA buffer. Proteins bound to the beads were eluted into 100 μL of SDS-PAGE sample buffer and analyzed by immunoblot.

### 4.9. Cell Surface Biotinylation

SERT surface expression was measured using a membrane-impermeant biotinylation reagent sulfo-NHS-SS-biotin as described previously [76]. In brief, cells stably expressing SERT WT or its mutants were treated twice with 1.0 mg/mL sulfo-NHS-SS-biotin in PBS for 30 min on ice. After rinsing with 100 mM glycine in PBS for 20 min on ice to quench excess sulfo-NHS-SS-biotin, cells were lysed, and the biotinylated proteins were captured by streptavidin-agarose beads at 4 °C overnight with gentle rotation. The biotinylated membrane proteins were eluted into 80 µL of SDS-PAGE sample buffer with 100 mM DTT at 22 °C for 30 min and analyzed by immunoblot.

### 4.10. Immunoblot

Protein samples were separated by electrophoresis on a 10% SDS-polyacrylamide gel and subsequently transferred onto a polyvinylidene difluoride membrane. After blocking with 5% non-fat milk in Tris-buffered saline containing 0.1% Tween-20, the membrane was incubated with a primary antibody overnight at 4 °C. The primary antibodies used in the study were a rabbit monoclonal anti-FLAG antibody (Proteintech, 1:10,000) for detecting the C-terminal FLAG-tagged SERT and a polyclonal anti-sGCβ1 antibody (Cayman, 1:500 dilution). After 3× washing with Tris-buffered saline, the membrane was incubated with an appropriate horseradish peroxidase-conjugated secondary antibody, and immunoreactive bands were visualized by SuperSignal West Pico with an eBlot Touch Imager (eBlot, Shanghai, China). The immunoreactive bands were then analyzed using ImageJ.

### 4.11. Statistical Analysis

All data were derived from experiments replicated a minimum of three times. Values are expressed as mean ± SEM. Asterisks indicate significance at a *p* < 0.05. All statistical analyses were performed using a *t*-test between two groups or one-way ANOVA between multiple groups.

## 5. Conclusions

Under physiological conditions, SERT activity is regulated by various modulators, including the physical protein–protein interactions, to maintain 5-HT homeostasis. However, life challenges frequently interfere with the regulation by altering SERT subcellular distribution or catalytic function, thereby leading to dysfunction of SERT and abnormal 5-HT signaling. In the present study, we developed two optogenetic approaches for exploring the cellular mechanism by which the SERT-sGC interaction regulates SERT function. Our results indicated that the regulation of SERT by the SERT-sGC interaction is through modulating SERT subcellular distribution rather than its catalytic function. In addition, the light-induced subcellular redistribution of SERT was shown to be attributable to an altered SERT membrane trafficking but not PKC-mediated internalization. Furthermore, the light-induced disruption of the SERT-sGC interaction increased SERT activity by enhancing its SERT cell surface expression. The present study provides not only insights into the mechanism underlying the regulation of SERT by the SERT-sGC interaction, but also an optogenetic platform to study the implication of 5-HT signaling in the pathophysiology of 5-HT-related psychiatric disorders or to develop a potential treatment approach by optogenetically manipulating the SERT-sGC interaction.

## Data Availability

The original contributions presented in this study are included in the article/Supplementary Materials. Further inquiries can be directed to the corresponding author.

## Supplementary Materials

The following supporting information can be downloaded at: https://www.mdpi.com/article/10.3390/ijms27104587/s1.

## Abbreviations

The following abbreviations are used in this manuscript:

5-HT	Serotonin
SERT	Serotonin transporter
sGC	Soluble guanylate cyclase
DRN	Dorsal raphe nucleus
IL4	The fourth internal loop
iLID	Improved light-induced dimer
CNS	Central nervous system
MDD	Major depressive disorder
SSRIs	Selective serotonin reuptake inhibitors
DAT	Dopamine transporter
NET	Norepinephrine transporter
PKG	cGMP-dependent protein kinase
nNOS	Neuronal nitric oxide synthase
APP^+^	4-(4-(dimethylamino)phenyl)-1-methylpyridinium
ER	Endoplasmic reticulum
