# Supplementary material for: Optogenetic Regulation of Localization and Function of Serotonin Transporter by Modulating Its Interaction with Soluble Guanylate Cyclase
Source: Int J Mol Sci. 2026 May 20;27(10):4587. doi: 10.3390/ijms27104587 (PMC13206950; doi:10.3390/ijms27104587)
Supplement: Supplementary file 1 [file ijms-27-04587-s001.zip › ijms-4246469-supplementary.pdf]

Supplementary Materials

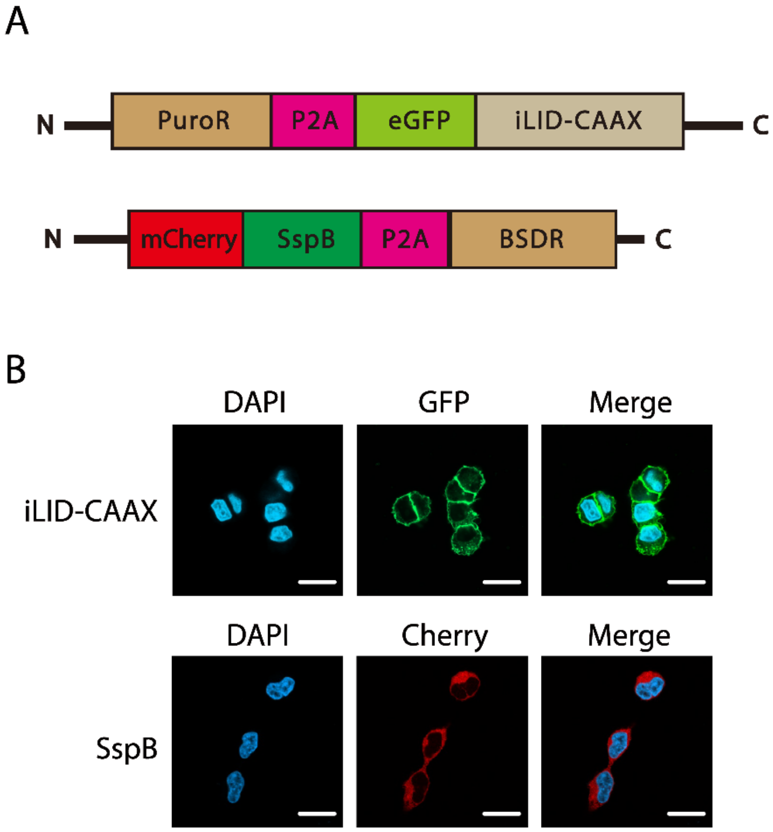

Figure S1. Subcellular localization of mCherry-SspB and eGFP-iLID-CAAX. (A) Viral constructs for generating stale cell lines expressing mCherry-SspB or eGFP-iLID-CAAX. (B) Representative confocal images of mCherry-SspB or eGFP-iLID-CAAX. Scale bars, 20 μm.

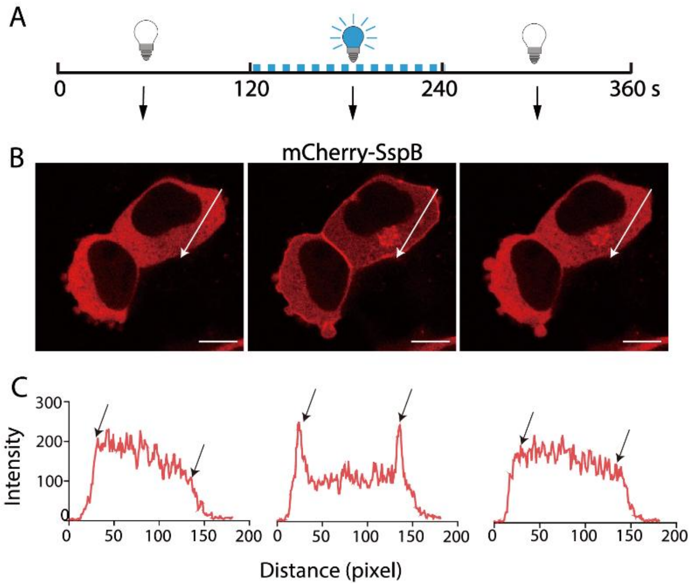

Figure S2. Effect of blue light illumination on mCherry-SspB localization in the plasma membrane. (A) Blue light was illuminated from 120 to 240-second in the light stimulation procedure. (B) Representative images of mCherry-SspB fluorescence were captured at 60, 180, and 300-second time points. Scale bars represent 20 μm. White line arrows represent the direction and place where mCherry fluorescence intensity was recorded. (C) Fluorescence intensity changes in the cells. The images were from confocal imaging of a representative cell during the light illumination procedure. Total eight cells in different sections were analyzed.

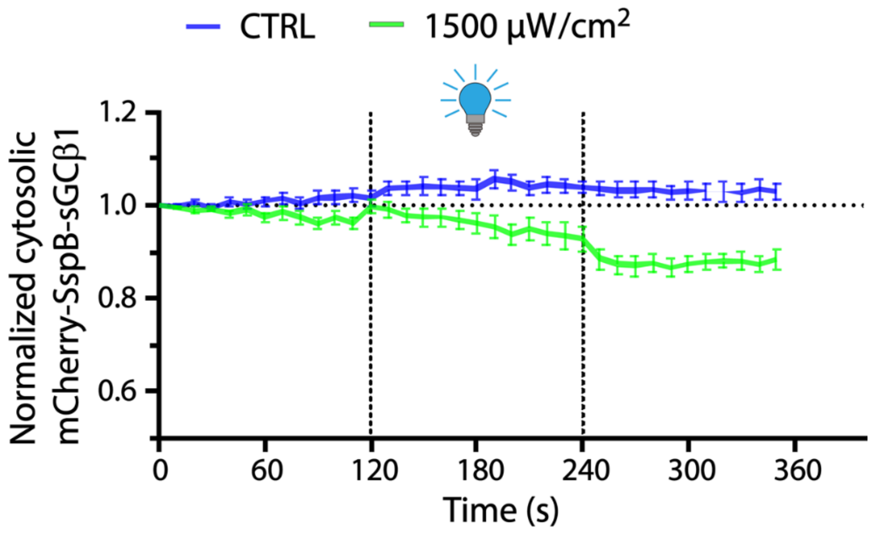

Figure S3. Time course of the cytosolic mCherry-SspB-sGCβ1 fluorescence in a 360-second light stimulation procedure. Blue light at 1500 µW/cm^2 was delivered from 120 to 240-second in the illumination procedure. CTRL, control measured without light illumination. Error bars represent ± SEM. Total eight cells were analyzed for alterations in the cytosolic fluorescence (n = 8).

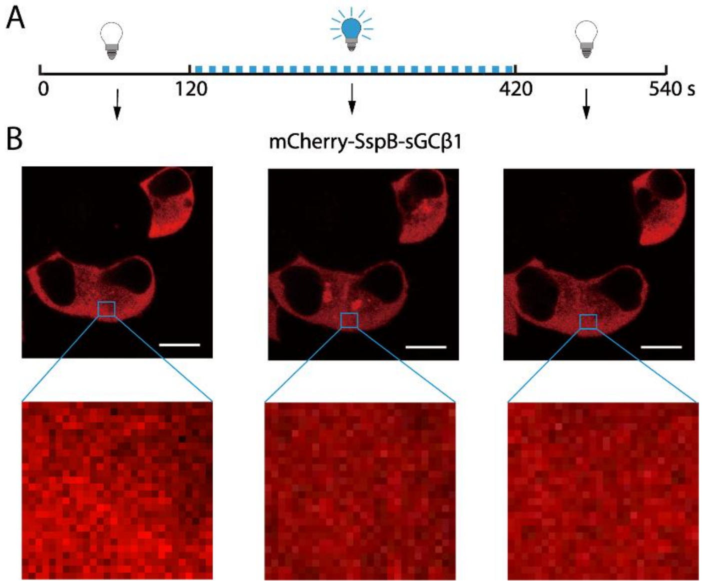

Figure S4. Blue light-induced decrease in the cytosolic mCherry-SspB-sGCβ1 fluorescence. (A) Schematic diagram of blue light illumination. Blue light illumination was performed during a time period from 120 to 420 seconds. (B) Representative images of the mCherry-SspB-sGCβ1 fluorescence were captured at 60, 270, and 480 seconds in the light stimulation procedure, respectively. Blue squares represent cytosolic areas selected for measuring fluorescence intensity. Scale bars, 20 μm. The images were from confocal imaging of a representative cell during the light illumination procedure. Total eight cells in different sections were analyzed.

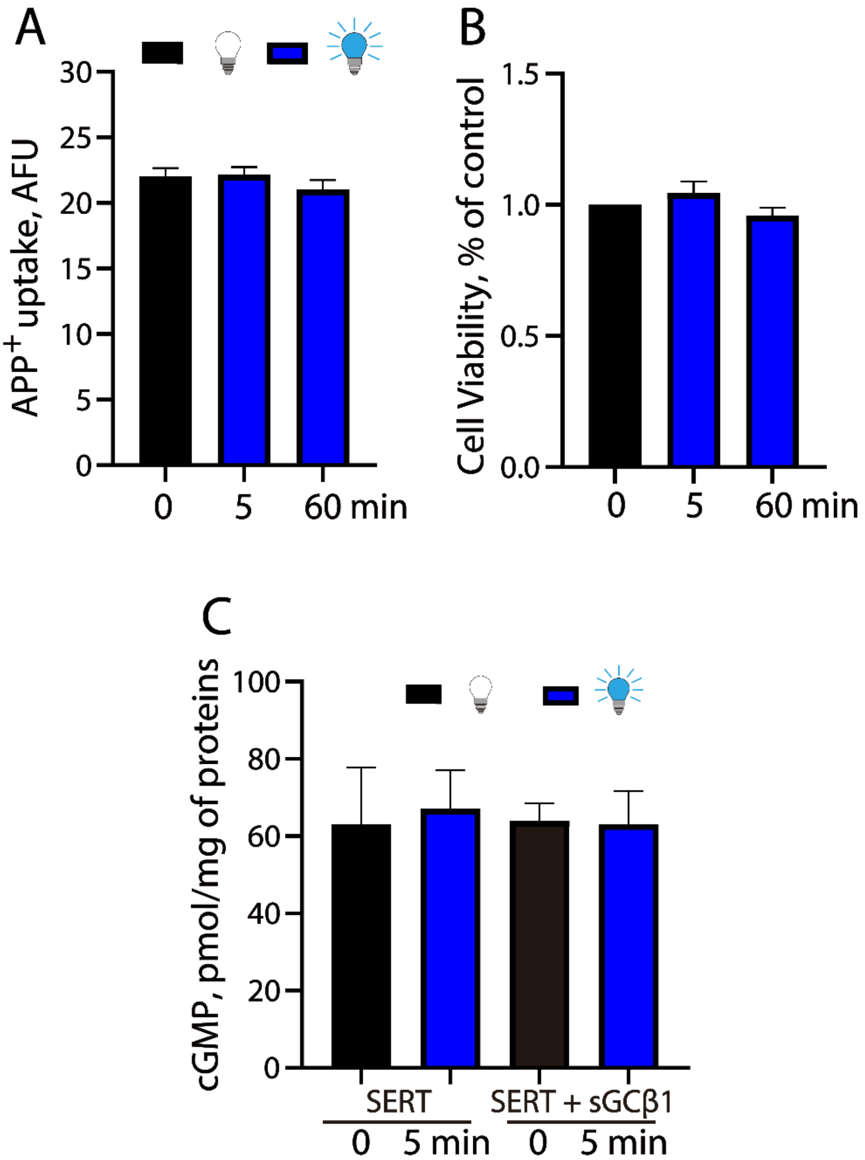

Figure S5. Effects of blue light illumination on APP^+ uptake, cell viability, and sGC activity. Cells stably expressing SERT or co-expressing SERT and sGCβ1 were treated with or without blue light illumination for 5 or 60 min and then used for analyzing for APP^+ uptake (A), cell viability (B), or sGC activity (C). For transport assay, cells were incubated with 2 μM APP^+ at 22 °C for 5 min and APP^+ fluorescence accumulated in the cells was measured. Cell viability was measured with a CCK-8 kit. For sGC activity assay, cells were pretreated with or without 100 μM SNAP at 22 °C for 15 min and cGMP production was then measured with cell lysates by ELISA. These assays were performed three times (n = 3). Error bars represent ± SEM.

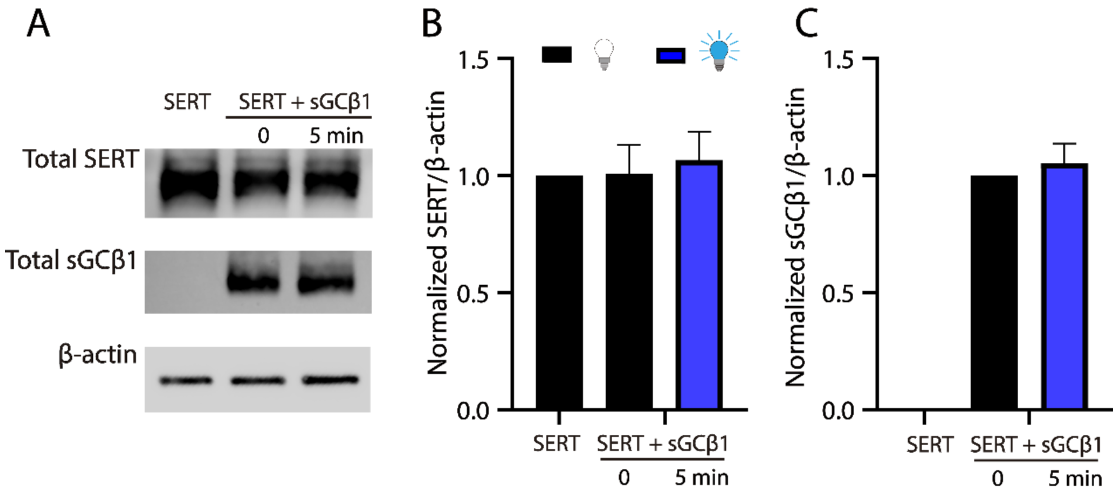

Figure S6. Total expression of SERT and sGC in the stable cell lines with or without light illumination. Cells stably expressing SERT or co-expressing SERT and sGCβ1 were treated with or without blue light illumination at 22 °C for 5 min and then lysed. The cell lysates were used for analyzing total expression of SERT or sGCβ1 with immunoblotting. These assays were performed three times (n = 3). Error bars represent ± SEM.
